# Wrist-Based Accelerometer Cut-Points to Identify Sedentary Time in 5–11-Year-Old Children

**DOI:** 10.3390/children5100137

**Published:** 2018-09-26

**Authors:** Jessica Chandler, Michael Beets, Pedro Saint-Maurice, Robert Weaver, Dylan Cliff, Clemens Drenowatz, Justin B. Moore, Xuemei Sui, Keith Brazendale

**Affiliations:** 1 College of Nursing, Medical University of South Carolina, Charleston, SC 29425, USA; 2 Department of Exercise Science, University of South Carolina, Columbia, SC 29208, USA; beets@mailbox.sc.edu (M.B.); weaverrg@mailbox.sc.edu (R.W.); msui@mailbox.sc.edu (X.S.); brazendk@email.sc.edu (K.B.); 3 Division of Cancer Epidemiology and Genetics, National Cancer Institute (NIH), Rockville, MD 20850, USA; pedro.saintmaurice@nih.gov; 4 School of Education, University of Wollongong, Wollongong, NSW 2522, Australia; dylanc@uow.edu.au; 5 Division of Physical Education, University of Education Upper Austria, Kaplanhofstrasse 40, 4020 Linz, Austria; clemens.drenowatz@ph-ooe.at; 6 Department of Family & Community Medicine, Wake Forest School of Medicine, Winston-Salem, NC 27101, USA; jusmoore@wakehealth.edu

**Keywords:** wearables, physical activity, measurement, youth, sedentary behavior, cut-points

## Abstract

Background: The objective of this paper is to derive a wrist-placed cut-point threshold for distinguishing sedentary behaviors from light-intensity walking using the ActiGraph GT3X+ in children. Methods: This study employed a cross-sectional study design, typically used in measurement-related studies. A sample of 167 children, ages 5–11 years (mean ± SD: 8.0 ± 1.8 years), performed up to eight seated sedentary activities while wearing accelerometers on both wrists. Activities included: reading books, sorting cards, cutting and pasting, playing board games, eating snacks, playing with tablets, watching TV, and writing. Direct observation verified sedentary behavior from light activity. Receiver operator characteristic (ROC) analyses were used to determine optimal cut-point thresholds. Quantile regression models estimated differences between dominant and non-dominant placement. Results: The optimal cut-point threshold for the non-dominant wrist was 203 counts/5 s with sensitivity, specificity, and area under the curve (AUC) of 71.56, 70.83, and 0.72, respectively. A 10-fold cross-validation revealed an average AUC of 0.70. Statistically significant (*p* ≤ 0.05) differences in median counts ranging from 7 to 46 counts/5 s were found between dominant and non-dominant placement in five out of eight sedentary activities, with the dominant wrist eliciting higher counts/5 s. Conclusion: Results from this study support the recommendation to place accelerometers on the non-dominant wrist to minimize “noise” during seated sedentary behaviors.

## 1. Introduction

Physical activity (PA) promotion in youth is a public health priority, as the incidence of childhood obesity remains alarmingly high. In order to determine progress towards meeting national PA guidelines, the ability to accurately measure progress towards guidelines is imperative. Accurate measurement of PA includes the ability to precisely distinguish PA from time spent sedentary, therefore, when new methodology is created and validated, there must be adequate attention towards classification of both physical activity and time spent sedentary.

Measurement of physical activity and sedentary behavior is evolving. Recently, the National Health and Nutrition Examination Survey (NHANES) discontinued the use of the previously validated, hip-mounted ActiGraph GT3x+ to measure physical activity and time spent sedentary, replacing it with the wrist-mounted ActiGraph GT3x+ [1]. Improved user compliance, an improved ability to measure sleep patterns, as well as the capability of capturing more upper extremity movements that may not have been captured using a device mounted at the hip were all considerations for moving the device to the wrist [2,3,4].

Because of this change, the device’s commonly used activity “count” output must be calibrated, and validity of data reduction approaches should be established using the new placement location (i.e., wrist) [5,6]. While both the NHANES and ActiGraph’s protocol suggest that the monitor be placed on a person’s non-dominant wrist, researchers have begun collecting data without evidence informing which wrist placement yields more accurate assessments of physical activity or time spent sedentary [7,8,9,10]. The non-dominant wrist has been proposed to ensure that activity counts collected during sedentary behaviors that involve upper extremity movement are not misclassified as physical activity (e.g., coloring, writing, playing with mobile electronic devices). Placement of the ActiGraph GT3x+ on the dominant wrist may misclassify time spent sedentary as time spent physically active. This issue is especially relevant to distinguishing light physical activity (LPA) from time sedentary, as it is the activity intensity adjacent to sedentary.

Sedentary behavior in children is defined by energy expenditure (EE) of 2.0 metabolic equivalent units (METS) or below [11], or by posture: in the lying down or sitting position [12]. Many sedentary activities in youth include large amounts of hand movement with little to no movement occurring at the hip. Examples of this include writing/coloring, videogames, board games, and most schoolwork. Placement of an activity monitor on the dominant wrist, therefore, is likely to result in increased activity counts for metabolically insignificant movements, such as writing, coloring, playing video games, watching television, reading, arts and crafts, etc. Recent calibration studies have created wrist-based cut-point thresholds for determining what range of “activity counts” correspond to different activity intensities (i.e., sedentary, light, moderate, vigorous, and moderate-to-vigorous intensities) [7,13]. Yet, both calibration studies and other recent research have included limited sedentary activities that require very little upper extremity movement [7,11,13]. Restricting activities to include such limited movement of the upper extremities is likely to have resulted in an underrepresentation of the range of counts/5 s for seated sedentary behaviors of elementary-aged children.

Empirical evidence for best practices related to placement of the wrist-worn ActiGraph GT3X+ and for defining sedentary behavior needs to be collected to better inform recommendations related to monitor usage and placement and data reduction. Thus, the purpose of this study is to examine differences between counts produced from accelerometers placed on dominant and non-dominant wrists during commonly performed seated sedentary activities that require upper extremity movement among 5–11-year-old children, and to determine optimal cut-point thresholds to distinguish sedentary behavior from light physical activity.

## 2. Data and Methods

A sample of 171 children between the ages of 5 and 11 years were recruited from one YMCA summer camp in Columbia, SC, to take part in this study. Children were eligible to participate in the study if they had no physical limitations that restricted their upper or lower body movements and could walk without an assistive device. The sample consisted of 58% male/42% female, 70% white/30% black, with a mean age 8.0 ± 1.8 years. Four participants’ data were deemed incomplete due to accelerometer failure and inability to identify dominant handedness on separate occasions, resulting in a total sample of 167 children. Participants self-reported their age and dominant hand. All children between the ages of 5 and 11 received an IRB-approved recruitment flyer upon summer camp registration containing the purpose of and rationale behind the current study. Parents contacted study staff if they either wanted to learn more or enroll their child(ren). At that point, study staff reviewed the informed consent document with the parent and answered any questions. Each child’s parent provided consent and each child gave verbal assent prior to each day of data collection. The University of South Carolina Institutional Review Board approved all methods: Approval Number PRO00043501

Data collection took place at a YMCA summer camp location over a 4-week period. The testing protocol lasted between 45 and 60 min per data collection session, and on average, three sessions were conducted each day. Seated sedentary activities included: reading books, playing/sorting cards, cutting and pasting from magazines, playing board games, eating a snack, playing games on a tablet, watching TV, and writing with a pencil. Walking served as the light-intensity activity from which all sedentary activities were distinguished. The children walked at a researcher-led, slow pace around a flat gymnasium floor. A description of all activities is listed in Table 1.

Prior to data collection, all accelerometers were initialized to begin and end recording data at the same time from the same computer using Eastern Standard Time. At the beginning of each data collection session, participants were fitted with two ActiGraph GT3X+ accelerometers, one on each wrist. In order to match accelerometer data with the correct participant and corresponding wrist (i.e., dominant vs. non-dominant), each accelerometer had a unique numeric identifier that was recorded alongside the participant’s name and demographic information. Consistent with previous protocols [5,11], each activity was performed for 5 min with 1-min breaks between activities for research assistants to set up the next activity (e.g., remove scissors and glue from the cutting/pasting portion and place pencil and paper for writing). During the 1-min rest breaks, children were instructed to stay seated and refrain from touching any materials being set up for the next activity until the research assistant said to begin. Each session included from three to five activities (mean of 4 activities per session) depending upon time available, and children performed an average of six unique activities ranging from three to nine performed activities. All participants in a data collection session completed the same set of activities at the same time. For instance, if a session of 15 participants included snack, board games, watching TV, and walking, all 15 participants in that session started and completed each individual activity together prior to moving on to the next activity. Trained research assistants were present during all sessions to indicate those participants that did not finish an activity session. Reasons for incomplete activities include bathroom breaks, dismissals, behavioral problems, and/or tampering with the accelerometers.

Direct observation was used to verify seated sedentary behaviors and walking [14]. Verification of sedentary behavior was defined as participants remaining in a seated position in a chair for the entirety of the activity. Data from children who stood up and took steps during the sedentary portion of a session were excluded from analysis for that seated sedentary behavior. However, if the child completed four of five seated sedentary behaviors, those four behaviors were included; only the activity during which the child was not seated was excluded from data analysis. Light activity intensity was verified when a child was walking at the researcher-set pace during the walking portion of the session [14].

The ActiGraph GT3X+ data were downloaded using ActiLife software (version 6.11.8) in 5-s epochs. All data was then transferred and processed using Stata/SE 13.1 software (StataCorp LP, College Station, TX, USA). All analyses were performed on axis 1, and we chose not to analyze the vector magnitude unit, as it did not result in better classification accuracy compared to axis 1 in our previous work; therefore, maintaining consistency in units allows for greater comparisons across studies [13]. Quantile regression models were used to compare the distribution of axis 1 counts produced at each wrist at the 25th, 50th, and 75th percentiles of the distribution of counts/5 s for each seated sedentary behavior and walking, separately. The 95% confidence intervals were estimated using 100 bootstrap samples, clustered based on each child to account for observations nested within children. A 10-fold receiver operator curve (ROC) analysis was conducted to derive the optimal cut-point thresholds for the dominant and non-dominant wrist to distinguish activity counts produced during seated sedentary behavior from light physical activity. The ROC analyses were conducted using data from the seated sedentary activity that produced the highest counts/5 s and data from walking, under the assumption that creating cut-points from the highest seated sedentary values would undoubtedly classify the activities producing lower values as sedentary. The ROC curves were calculated by dichotomizing intensities, then determining the cutoff value that maximized both sensitivity and specificity. This procedure was performed 10 times for both the dominant and non-dominant wrist. The cut-point threshold, sensitivity, specificity, and area under the curve (AUC) values produced for each of the 10 ROC iterations were averaged to derive the final values for each wrist placement.

## 3. Results

The sample of participants were 58% male/42% female, 70% white/30% black, mean age 8.0 ± 1.8 years. During analysis, four participants were excluded for incomplete data, resulting in a final sample size of 167 participants aged 5–11 years. Descriptive statistics of axis 1 counts per 5 s epoch (counts/5 s) during each activity on both wrists are presented in Table 2.

Watching television consistently produced the lowest counts/5 s, while playing board games was the seated sedentary activity that produced the highest counts/5 s. Results from the quantile regression models are also presented in Table 2. There were significant differences in the median counts/5 s between the non-dominant and dominant wrist placement during board games, cards, and cutting and pasting. Further, when analyzing the 75th percentile, differences in counts/5 s during writing were observed. The largest difference was observed for board games, with the median counts/5 s from the dominant wrist 46 counts/5 s higher than the non-dominant. The smallest difference was 1 count/5 s during reading. Across all activities, counts/5 s on the dominant wrist were higher than those on the non-dominant.

The distribution of counts/5 s for each seated sedentary activity compared to walking for dominant and non-dominant placements are presented in Supplementary Figure S1. There was a rightward shift, indicating a higher distribution of higher counts/5 s of the dominant wrists’ distribution during cards, cut/paste, games, and tablets compared to the non-dominant wrist. This is consistent with the quartile regression results that indicate the dominant wrist produces higher counts/5 s during all seated sedentary activities.

Cut-point thresholds with corresponding sensitivity, specificity, and area under the curve (AUC) data are listed in Table 3. The optimal cut-point thresholds for the dominant wrist are all higher compared to those derived for the non-dominant wrist. The AUC values, however, were higher for the non-dominant wrist (0.66–0.72) compared to the dominant wrist (0.60–0.68) placement, with axis 1 producing the highest and axis 3 producing the lowest AUC for each wrist.

## 4. Discussion

The findings from this study indicated that differences in counts/5 s exist between the non-dominant and dominant wrist placements during seated sedentary behaviors. Seated sedentary activities requiring upper extremity movement produced higher counts than those that require very little to no movement (e.g., playing board games vs. watching TV). During activities requiring movement, the dominant wrist produced higher counts/5 s compared to the non-dominant wrist, resulting in a higher cut-point threshold for distinguishing light physical activity from sedentary behavior. ROC analyses resulted in higher sensitivity, specificity, and AUC values for the non-dominant wrist, indicating that the dominant wrist more often misclassifies seated sedentary activities as light physical activity.

Playing with tablets, writing, board games, sorting cards, and cutting and pasting resulted in significantly higher counts/5 s from the dominant compared to the non-dominant wrist placement. The remaining activities (i.e., reading books, watching TV, and eating snacks) resulted in no differences. The lack of difference in counts was due to the fact that neither arm was moving, recording counts/5 s close to zero from both dominant and non-dominant wrist placements. For writing and playing games on tablets, results varied by percentile of counts/5 s. At the lowest quartile, no differences existed, while the middle and upper quartile revealed significant differences in counts/5 s between wrist placements. These results indicate the distribution of counts/5 s is not uniform, but when differences exist, the dominant wrist always produces higher activity counts. When the newly created cut-points were applied to previously collected child data from an 8 h period of summer camp, the non-dominant and dominant wrist-based cut-points resulted in 317 and 337 min spent sedentary, respectively. These findings have important implications for accelerometer wrist placement, as it is clear that the dominant wrist produces higher counts during seated sedentary activities that will more often be misclassified as LPA. Simply put, wrist placement of monitor is in fact an important consideration when designing a study and analyzing and distilling raw count data.

A recent study reported no differences in total daily activity counts between the dominant and non-dominant wrist in young adults [15]. These results, however, are not comparable to the current study for two reasons. The present study focused solely on difference in counts collected during time spent sedentary. During physical activity, both wrists are likely moving identically, as there are no differences in count/5 s distribution during walking, as illustrated in Supplementary Figure S1. Therefore, wrist placement may not be important during physical activity; however, for seated sedentary behaviors, differences exist. Though counts/day may have been similar in the previous study, the data were not distilled into activity intensities, so it remains unclear if each wrist placement’s estimate of time spent in discrete activity intensities would have been equivalent. Second, the previous study included young adults aged 18–35, and results may not be generalizable for children, as their movement patterns differ greatly from adults [15,16].

ROC analyses derived optimal cut-point thresholds of 202 and 221 counts/5 s for the non-dominant and dominant wrist placement, respectively. Both cut-point thresholds are higher, and AUC values lower, than previously reported [7,13]. This was not unexpected, as higher cut-point thresholds were anticipated, since the protocol intentionally included a battery of activities that required upper extremity movement while seated. The present study found AUC values of 0.72 and 0.67 for the non-dominant and dominant wrist placement, respectively, which is substantially lower than the AUC values reported in recent wrist-placed calibration of 0.89, 0.94, and 0.95 [7,13,17]. While the current study found lower AUC values than other wrist-based calibration studies, they are comparable to many widely cited hip-based cut-points [18]. Additionally, the current study included more realistic and active sedentary activities compared to previous wrist-based calibrations. Lower AUC values in the present study can be explained by the wider variety of seated sedentary activities requiring upper extremity movement that created a distribution of activity counts that overlapped with the distribution of activity counts from walking. Further, if all sedentary activities and corresponding counts/5 s had been included in the ROC analysis, higher sensitivity, specificity, and AUC values would have been produced. Even so, the newly derived cut-point thresholds can be used with confidence in capturing more accurate estimates of time spent sedentary in 5–11-year-old children compared to previously published wrist-based cut-points that may misclassify time spent sedentary as meaningful light physical activity [7,13,17].

There are several strengths of this study. First, a sample of 167 unique children is similar to or exceeds many of the widely cited calibration studies of hip-placed accelerometer in children [19,20,21]. Secondly, rather than including a majority of largely motionless seated sedentary behaviors, this study included a wide variety of seated sedentary activities involving the use both arms [7,13]. This was important because previously published wrist-based cut-points were derived using activities that were motionless or only utilized the one hand, thus resulting in cut-point thresholds that overestimate LPA and underestimate time spent sedentary. Lastly, the ROC analysis is a common approach in determining optimal cut-point thresholds, and the use of a 10-fold calibration and cross-validation strengthens the statistical methods used [17,18]. The study also has limitations. First, the protocol lacked a free-living component and was designed so that each activity started and ended at a certain time for an entire group of children. Free-living conditions in which children are free to choose the activities in which to partake may yield different data, and therefore should be tested. Secondly, not all children completed the full battery of nine activities because of time, location, and attendance restraints of the summer day camp. Even so, the range of child observations per sedentary activity ranged from 90 to 120, exceeding other hip-based calibration studies [19,20,21]. Lastly, the use of direct observation as a criterion measure for sedentary behavior may be criticized, however, with the current debate over metabolic equivalents indicating sedentary behavior [11], classification of sedentary activity by posture (i.e., seated) was deemed appropriate for the current study [12]. The use of direct observation allowed for verification that every participant included in data analysis was seated for all activities, regardless of MET values that might have otherwise classified seated sedentary activities as light PA.

## 5. Conclusions

The developed cut-point thresholds for each wrist placement can be used with confidence when distinguishing seated sedentary behavior from light-intensity walking and when measuring sedentary behavior as the primary outcome variable. The results from the current study suggest for the continued use of the non-dominant wrist placement for children in order to be consistent with already published suggestions and protocols for the ActiGraph GT3X+ and to minimize the misclassification of upper extremity movement during seated sedentary activities as light physical activity.

## Figures and Tables

**Table 1:** Description of performed activities.

Activity	Description
Reading	Age-appropriate books were provided for the children to choose from and read to themselves.
Cards	Children were given a portion of a deck of cards and asked to do several sorting tasks, such as highest to lowest, sort by suite, and sort by color.
Cut/Paste	Children were given magazines, scissors, glue sticks, and construction paper to create a collage.
Games	Board games and building blocks were given to the children to play with on tables.
Snack	Children were given the choice of a granola bar or applesauce with a spoon and asked to eat their snack and let a research assistant know when they were finished. Start time was the same for every child, but stop time was individual to when the child told the research assistant they were finished eating their snack.
Tablets	Tablets were provided for children to explore through activities. such as online games, typing facts about themselves, and searching the Internet.
TV	Children were provided with an age-appropriate movie to watch as a group.
Walking	All children walked alongside a research assistant at a pace to elicit light physical activity. The pace was led by the research assistant to ensure children did not move quicker than a light-intensity walk.
Writing	Children were given writing worksheets to trace letters and write complete sentences using a pencil.

**Table 2:** Descriptive statistics and quantile regression results testing differences in counts produced by dominant and non-dominant wrist placement (*n* = 167 unique children and 937 child observations).

		Counts/5 s Epoch				Quantile Regression					
		Dominant		Non-Dominant		25th Percentile		50th Percentile		75th Percentile	
	*n*	Median (IQR)		Median (IQR)		Diff	95% CI	Diff	95% CI	Diff	95% CI
TV	98	0	(0–2)	0	(0–0)	—	—	—	—	—	—
Tablets	96	14	(0–66)	0	(0–40)	—	—	**14**	(8.5, 19.5)	**26**	(16.0, 36.0)
Writing	98	7	(0–65)	0	(0–42)	—	—	**7**	(3.7, 10.3)	**23**	(11.3, 34.7)
Snack	100	20	(0–93)	12	(0–80)	—	—	8	(−1.3, 17.3)	13	(−2.9, 28.9)
Reading Books	97	36	(0–142)	35	(0–137)	—	—	1	(−8.8, 10.8)	5	(−7.3, 17.3)
Board Games	120	129	(29–246)	83	(0–210)	**29**	(16.3, 41.7)	**46**	(25.6, 66.4)	**45**	(25.7, 64.3)
Cards	110	106	(22–219)	74	(8–176)	**14**	(5.7, 22.3)	**32**	(16.6, 47.4)	**43**	(23.9, 62.1)
Cut/paste	90	85	(21–171)	68	(8–158)	**13**	(3.6, 22.4)	**17**	(5.3, 28.7)	**13**	(2.6, 23.4)
Walking	128	357	(193–607)	357	(195–602)	−2	(−19.0, 15.0)	−1	(−22.8, 20.8)	5	(−26.5, 36.5)

**Bold** text indicates significant difference between counts produced by dominant and non-dominant wrists, ‘—’ indicates lack of convergence during the quantile regression, IQR: Interquartile Range, CI: Confidence Interval.

**Table 3:** Optimal cut-point thresholds, sensitivity, specificity, and AUC based on ROC analyses.

	Calibration (n = 100)				Cross-Validation (n = 67)
	**Non-Dominant Hand**				
	**Counts/5 s**	**Sensitivity (%)**	**Specificity (%)**	**AUC (95% CI)**	**AUC (95% CI)**
Axis 1	203	71.6	70.8	0.72 (0.71–0.73)	0.70 (0.69–0.71)
Axis 2	200	66.6	69.7	0.71 (0.70–0.72)	0.70 (0.69–0.71)
Axis 3	201	65.1	62.5	0.66 (0.65–0.67)	0.65 (0.63–0.66)
VM	397	68.2	66.9	0.70 (0.69–0.71)	0.69 (0.68–0.70)
	**Dominant Hand**				
	**Counts/5 s**	**Sensitivity (%)**	**Specificity (%)**	**AUC (95% CI)**	**AUC (95% CI)**
Axis 1	229	67.1	66.8	0.67 (0.66–0.68)	0.68 (0.67–0.69)
Axis 2	220	64.0	62.0	0.64 (0.64–0.65)	0.65 (0.64–0.66)
Axis 3	219	60.8	58.8	0.60 (0.59–0.61)	0.60 (0.58–0.61)
VM	428	63.9	62.9	0.65 (0.64–0.65)	0.64 (0.63–0.66)

AUC: area under the curve, ROC: receiver operator characteristics, CI: confidence interval, VM: vector magnitude.

## Supplementary Materials

The following are available online at http://www.mdpi.com/2227-9067/5/10/137/s1, Figure S1: Distribution of counts/5 s epoch for each sedentary activity versus walking.
