# Supplementary material for: Wrist-Based Accelerometer Cut-Points to Identify Sedentary Time in 5–11-Year-Old Children
Source: Children (Basel). 2018 Sep 26;5(10):137. doi: 10.3390/children5100137 (PMC6210293; doi:10.3390/children5100137)
Supplement: Supplementary file 1 [file children-05-00137-s001.pdf]

Supplementary Figure 1. Distribution of counts/5second epoch for each sedentary activity versus walking

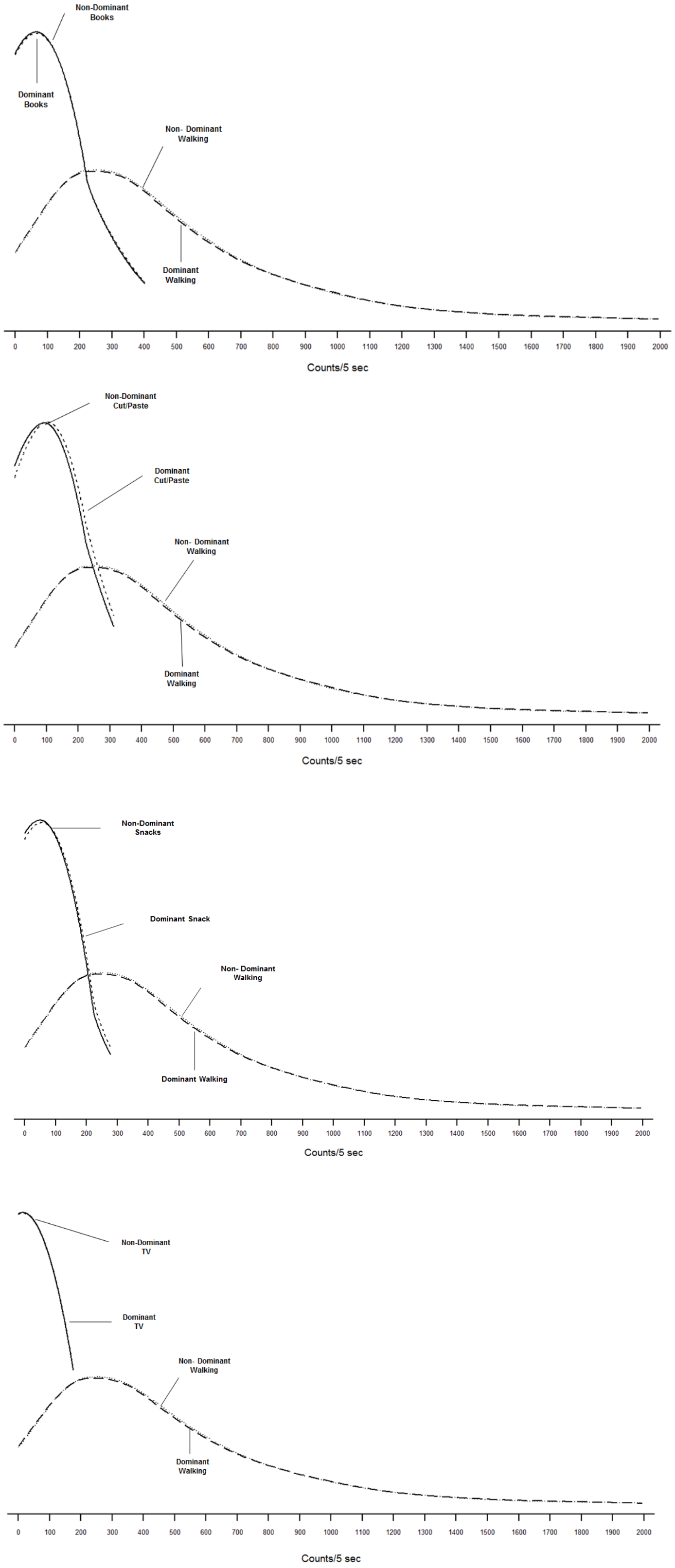


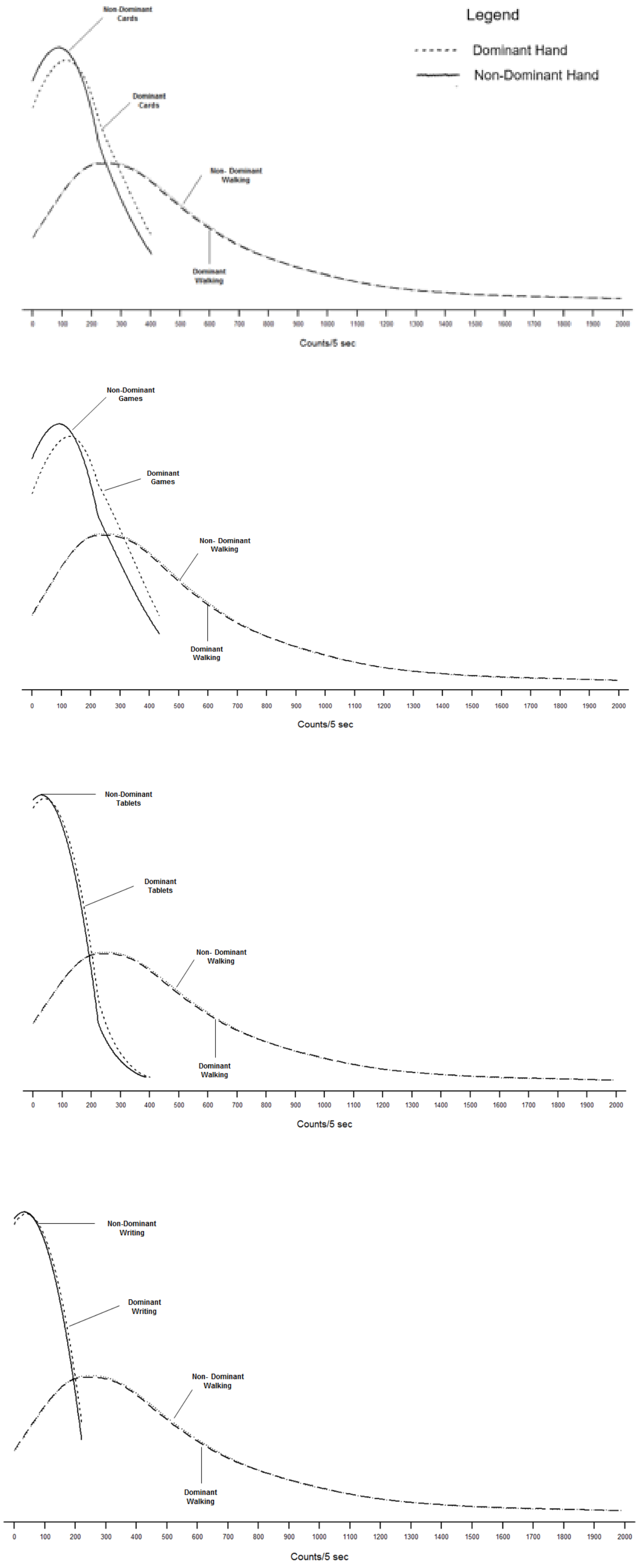


*All y-axes units are kernel density and these supplemental figures are meant to show where each activities mean count/5seconds would intersect with walking, separated by wrist placement*
